# Population based study on the progress in survival of primarily metastatic lung cancer patients in Germany

**DOI:** 10.1038/s41598-024-66307-3

**Published:** 2024-07-11

**Authors:** Therese Tzschoppe, Julia Ohlinger, Dirk Vordermark, Ahmed Bedir, Daniel Medenwald

**Affiliations:** Department of Radiation Oncology, Health Services Research Group, University Hospital Halle (Saale), Ernst-Grube-Str. 40, 06120 Halle (Saale), Germany

**Keywords:** Lung cancer, Germany, Cancer registry, Survival, Oncology, Cancer, Cancer epidemiology, Cancer therapy, Lung cancer, Metastasis, Medical research, Epidemiology, Outcomes research

## Abstract

Lung cancer is known for its high mortality; many patients already present with metastases at the time of diagnosis. The aim of this study is to assess the impact of new treatment strategies on the survival of primarily metastatic lung cancer patients and to analyze the differences in outcomes between non-small cell lung cancer (NSCLC) and small cell lung cancer (SCLC) patients. Population-based data, provided by the Robert-Koch Institute in Germany, was used and patients diagnosed between 2007 and 2018 were included in the study. We differentiated between NSCLC and SCLC patients and analyzed the survival over time for both sexes separately, using the Kaplan–Meier method. To evaluate survival advantages, we calculated multivariable hazard ratios. In total, 127,723 patients were considered for the study. We observed a moderate increase in survival over time. All patients showed an increased survival rate when undergoing chemotherapy. Minimal to no increase in survival was shown in NSCLC patients when receiving radiotherapy, whereas SCLC patients’ survival time did benefit from it. NSCLC patients receiving immunotherapy showed an increase in survival as well. It can be concluded that advancements in radiotherapy, the application of chemotherapy, and the introduction of immunotherapies lead to an increased survival time of both NSCLC and SCLC primarily metastatic lung cancer patients.

## Introduction

Lung cancer is the second most commonly diagnosed malignant neoplasm worldwide and the leading cause of cancer mortality^1^. In 2020 an estimated 1.8 million patients died of lung cancer worldwide^1^.

Among the many factors contributing to its high mortality, the lack of early symptoms and insufficient screening methods are two major reasons why most cases are not diagnosed until a later stage^2^. In Germany, over 50% of lung cancer cases are not diagnosed until reaching UICC-stage IV (Union for International Cancer Control)^2^. At this stage, the cancer has already metastasized into the contralateral lung or into at least one other organ, resulting in an unfavorable prognosis^3^: in 2016, the 5-year survival rate of primarily metastatic patients was estimated to be approximately 5%^4^. Hence, there is a need for reliable and innovative therapies, especially for this affected group of patients.

In the past 20 years, new approaches have emerged and therapy plans have drastically changed, resulting in an increased life expectancy and an improved quality of life^2^.

This downward trend of mortality has also been shown in epidemiological studies in Europe^5^.

Today, personalized medicine leads the way into the future of cancer therapy. This is especially important for patients with primarily metastatic lung cancer, who are the primary beneficiaries of immunotherapy and targeted therapies. In non-small cell lung cancer (NSCLC) patients diagnosed with stage IV cancer, an increase of up to four years of survival time can be expected^6^.

Furthermore, technological advances in radiotherapy have not only led to a reduction of incidental irradiation of surrounding healthy tissue but also enabled more precise targeting of tumors^7^. This has improved outcomes both in reducing toxicity and in increasing the survival rates of lung cancer patients^7^.

These and other new therapies continue to be introduced and adjusted; however, in order to evaluate the success of these therapies, population-based studies are required^8^. The aim of our study is to assess the impact of new lung cancer treatment strategies on survival of the primarily metastatic patient group over time and to analyze the differences in outcomes between NSCLC and small cell lung cancer (SCLC) patients.

Overall, we hypothesized that survival times would increase due to treatment advancements.

## Methods

### Data source

The data for this longitudinal, population-based study is provided by the Robert-Koch Institute (RKI), the German federal government agency coordinating preventive health care and disease control. The RKI collects federal data about the cancer patients which is anonymized and then published in the „German Epidemiological Cancer Registry“ for public use^9^. The dataset includes the recent numbers and characteristics of cancer patients in Germany^10^. The German Epidemiological Cancer Registry is a large representative medical database and its validity in cancer research has been proven^11^. The data undergoes frequent quality checks and is then pooled for analyses.

In order to analyze survival, we used the following patient-level information: the patient’s sex, the federal-state of residence, the age at diagnosis, the date of diagnosis and date of death as well as the received therapies. Information about the treatment is displayed as binary variables in the pooled cancer registries (surgery yes/no, chemotherapy yes/no, radiotherapy yes/no, immunotherapy yes/no).

Additionally we used the cancers International Classification of Diseases (ICD-10), its histology and TNM stage (TNM Classification of Malignant Tumors by the UICC, version 6 and 7) at diagnosis^12^.

The RKI data set includes cases that were diagnosed through an autopsy or were registered based on a death certificate. Since the data of these cases is irrelevant for this study, these cases were excluded.

Furthermore, following the recommendations from The European Cancer Registry-based Study on Survival and Care of Cancer Patients (EURO-CARE-5 Study)^13^, federal states who reported a high number of death certificate only (DCO) or autopsy only cases were identified and excluded as well. This step will prevent poor data quality. We decided to exclude federal states that reported more than 15% of their cases using DCO and autopsy methods.

### Study population

According to the classification of lung cancer by the World Health Organization (WHO)^14^, we distinguished in the analysis between small cell lung cancer (SCLC) and non-small cell lung cancer (NSCLC).

Patients were selected from the database by using the „International Statistical Classification of Diseases and Related Health Problems“ (ICD Code) of the WHO. The database works with its 10. Version. We included patients by using the ICD-10 Code C34 (malignant neoplasm of bronchia and lung) in the study.

To analyze the different histological groups of lung cancer, the morphology codes according to the WHO’s „International classification of diseases for oncology“ (ICD-O Code), 3. Version, were used. The ICD-O Codes that were taken into the analysis can be found in the appendix (Table S7). Histological subtypes with ICD-O Codes that provided less than 5 patients were excluded as well as patients with an ICD-O Code that suggests the patient’s neoplasm of the lung is of an uncertain origin.

To ensure that only primarily metastatic lung cancer patients are included in the analysis, the TNM staging was used. Only patients with the M-category (metastasis) M1 (metastases to distant organs, beyond regional lymph nodes) were chosen for the study. During the analyzed period (2007–2018), two new editions of the TNM-staging system were published^15,16^. While the changes of the staging system over time could result in biased outcomes, it is important to note that the M-category has remained mostly unaffected. The 7th edition in 2009 added the two subcategories M1a and M1b^16^. In 2018, with the TNM’s 8th edition, subcategory M1c was added^15^. The study therefore includes all cases of M1, M1a, M1b and M1c.

Four time-periods (2007–2009, 2010–2012, 2013–2015 and 2016–2018) were defined in order to evaluate the effects of the different cancer treatments over time. The patients were arranged into groups based on their year of diagnosis.

The cases were all censored in December 2019 as it marks the end of the observation period of this study. Additionally, to avoid potential bias, cases were censored after a follow-up period of 36 months (approximately 3 years). Particularly, it addresses the potential variability in survival outcomes, as patients diagnosed in more recent years might have experienced more favorable outcomes compared to those diagnosed earlier in the observation period.

### Databank search and case identification

The RKI Cancer Registry provided a total of 323,556 lung cancer cases diagnosed between 2007 and 2018. 142,092 of these patients already had metastases at the time of diagnosis.

Due to the exclusion of federal states that provided more than 15% of their cases through DCO and autopsy only, patients from Schleswig–Holstein, Nordrhein-Westphalen (North Rhine-Westphalia), Hessen (Hesse), and Sachsen-Anhalt (Saxony-Anhalt) were not included in our analysis.

The search yielded 26,804 primarily metastatic SCLC patients and 95,060 primarily metastatic NSCLC patients, from which, 63,263 patients had the diagnosis adenocarcinoma, 20,584 squamous cell carcinoma, and 3008 large cell carcinoma.

### Statistical analysis

We calculated the median overall survival (OS) and the 3-year OS, first for the entire length of the study, then separately for the four time-periods. The 3-year survival rates of patients who received chemotherapy, radiotherapy, or immunotherapy were compared to those of patients who did not receive these therapies, respectively.

The 3-year survival rates were illustrated using the Kaplan–Meier method.

A log-ratio test was performed to measure the significance of the Kaplan–Meier estimates using a set significance level of 5%.

Multivariable hazard Cox-regression models were used to analyze the association of grouped parameters with the patient’s mortality. The potential impact contributed by cancer-related parameters known to influence the overall survival between the time-periods was assessed by entering these factors sequentially into our Cox-regression models. Our crude model was only adjusted for the included time-periods. We further adjusted for age, received therapy (chemotherapy, radiotherapy, immunotherapy), histological grade, and tumor characteristics (such as tumor size and lymph node involvement) in our models. The computed hazard ratios (HR) with 95% confidence intervals (CI) were calculated using the time-period 2007–2009 as the reference group. The analysis was stratified for NSCLC and SCLC. Both groups were further stratified according to the patient’s sex. To make sure that there are no interactions between the variables and time-periods, we calculated several hazard ratios.

All statistical analyses were performed using R version 2023.06.1^17^.

### Bias and sensitivity analysis

To ensure the robustness of the estimated HRs, we performed several sensitivity analyses.

In Western Germany, only a few federal states started contributing to the German Epidemiological Cancer Registry in early the 2000s, with Bayern (Bavaria) and Nordrhein-Westphalen (North Rhine-Westphalia) joining in 2002 and 2006, respectively^18^. In contrast, Eastern Germany has a longer history of tracking medical registries, dating back to 1952^18^. This discrepancy raises concerns about potential variations in case recording practices across the different registries, possibly leading to bias in the study's outcomes. To address this and prevent any potential bias, we compared the HRs from West Germany with those from East Germany.

During the analyzed time-periods the application of Positron Emission Tomography (PET) for correct staging of lung cancer patients has continuously grown. The combined use of the functional data of PET and the anatomic data of the computer tomography (CT) brought many advantages and led to more precise staging, resulting in more appropriate treatment approaches, and ultimately improving the prognosis of the patients^19^. With regards to our study, the increasing use of the PET/CT means that patients are more likely to be diagnosed with metastases in other organs as well. Since only stage IV lung cancer patients were included in our study, this could potentially result in a biased situation. In order to evaluate the resulting effect, we analyzed the percentage of stage IV lung cancer patients from all lung cancer patients over time in the dataset provided by the RKI.

Furthermore, we performed an analysis to check for interaction effects between different treatments. Specifically, we included an interaction term in our Cox proportional hazards models to evaluate whether the combined effect of these treatments on survival differed from their individual effects.

## Results

### Patient characteristics

In total, 127,723 cases were considered for the study. The mean age of diagnosis for patients with primarily metastatic lung cancer included in this study is 67.3 years (Table 1).

**Table 1:** Characteristics of patients diagnosed with primarily metastatic lung cancer 2007–2018.

Characteristics	Total	Period 2007–2009	Period 2010–2012	Period 2013–2015	Period 2016–2018
**All patients**	127 723 (100%)	22 862 (18%)	31 475 (24.5%)	36 755 (29%)	36 631 (28.5%)
Male	82 642 (64.7%)	15 568	20 845	23 417	22 812
Female	45 081 (35.3%)	7 294	10 630	13 338	13 819
Mean age at diagnosis, years ± SD	67.3 ± 10.4	66.3	67.0	67.5	68.0
Male ± SD	67.7 ± 10.1				
Female ± SD	66.6 ± 10.9				
**SCLC patients**	26 804 (100%)	5 029 (19%)	6 617 (24.5%)	7 666 (28.5%)	7 492 (28%)
Male	17 262 (64.4%)	3 419	4 363	4 847	4 633
Female	9 542 (35.6%)	1 610	2 254	2 819	2 859
Mean age at diagnosis, years ± SD	66.9 ± 9.7	65.9	66.7	67.1	67.5
Male ± SD	67.4 ± 9.7				
Female ± SD	66.0 ± 9.6				
**NSCLC patients**	95,060 (100%)	16,564 (17%)	23,413 (25%)	27,576 (29%)	27,507 (29%)
Male	61,658 (65%)	11,289	15,549	17,639	17,181
Female	33,402 (35%)	5275	7864	9937	10,326
Mean age at diagnosis, years ± SD	67.2 ± 10.5	66.2	66.9	67.4	67.9
Male ± SD	67.6 ± 10.1				
Female ± SD	66.5 ± 11.1				
Adenocarcinoma	63,263 (100%)	9905 (16%)	15,290 (24%)	18,851 (30%)	19,217 (30%)
Male	38,006 (60%)	6233	9378	11,213	11,182
Female	25,257 (40%)	3672	5912	7638	8035
Mean age at diagnosis, years ± SD	66.6 ± 10.8	65.3	66.1	66.8	67.4
Male ± SD	67.0 ± 10.4				
Female ± SD	66.1 ± 11.3				
Squamous cell carcinoma	20,584 (100%)	4326 (21%)	5114 (25%)	5708 (28%)	5436 (26%)
Male	16,073 (78%)	3457	4084	4437	4095
Female	4511 (22%)	869	1030	1271	1341
Mean age at diagnosis, years ± SD	68.9 ± 9.6	67.9	68.7	69.2	69.5
Male ± SD	69.0 ± 9.5				
Female ± SD	68.6 ± 9.8				
Large cell carcinoma	3008 (100%)	678 (23%)	895 (30%)	763 (25%)	672 (22%)
Male	2071 (69%)	471	618	538	444
Female	937 (31%)	207	277	225	228
Mean age at diagnosis, years ± SD	66.5 ± 10.5	65.0	66.7	66.7	67.3
Male ± SD	66.9 ± 10.2				
Female ± SD	65.4 ± 11.3				

*SD* standard deviation.

SCLC patients were, on average, diagnosed at 66.9 years of age and NSCLC patients at 67.2 years of age. For all studied entities, the mean age of diagnosis for male patients was slightly higher than female patients. Patients diagnosed with primarily metastatic large cell carcinoma and adenocarcinoma had the lowest mean age of diagnosis (66.5 and 66.6 years).

A trend toward diagnosis at an older age in more recent time-periods can be observed. A total of 82,642 men and 45,081 women were diagnosed. In all four time-periods the majority of patients were male (64.7%), though the number of women (35.3%) diagnosed with NSCLC and SCLC has continuously risen in the observed time-periods. The group diagnosed with primarily metastatic adenocarcinoma had the highest percentage of female patients (40%).

Approximately 74.4% of the patients were diagnosed with NSCLC and 25.6% with SCLC.

The amount of diagnosed lung carcinomas grew progressively over time (the only exception being large cell carcinoma and squamous cell carcinoma in the last time-period). A more precise documentation of the diagnosed histologies over time shown as percentages of the period’s total patients can be found in the appendix (Table S8).

### Treatment patterns

We stratified the patients’ 3-year survival rates according to the cancer’s histology, time-period, and treatment modality (Table 2).

**Table 2:** 3-year survival of patients diagnosed with primarily metastatic lung cancer 2007–2018, stratified by the cancers histology, time-period and treatment modality: significant results are portrayed in bold numbers.

	NSCLC adenocarcinoma 3-year survival, % (95% CI)		NSCLC squamous cell carcinoma 3-year survival, % (95% CI)		NSCLC Large cell carcinoma 3-year survival, % (95% CI)		SCLC 3-year survival, % (95% CI)	
	Therapy received	Therapy not received	Therapy received	Therapy not received	Therapy received	Therapy not received	Therapy received	Therapy not received
Chemotherapy								
2007–2009	13.2% (12.2–14.2)	11.9% (10.8–13.2)	10.1% (8.8–11.6)	7.8% (6.5–9.4)	11.2% (7.9–15.7)	8.1% (5.2–12.7)	7.2% (6.2–8.2)	5.6% (4.4–7.3)
2010–2012	12.0% (11.3–12.8)	11.2% (10.4–12.2)	9.2% (8.1–10.5)	8.3% (7.1–9.7)	9.0% (6.6–12.2)	7.9% (5.4–11.5)	6.3% (5.6–7.2)	4.7% (3.7–6.0)
2013–2015	**14.3% (13.5**–**15.1)**	**12.0% (11.2–12.8)**	**11.3% (10.1–12.7)**	**7.5% (6.4–8.8)**	6.8% (4.6–9.9)	6.0% (3.6–10.0)	**7.3% (6.5–8.2)**	**4.6% (3.6–5.8)**
2016–2018	19.2% (18.1–20.4)	17.6% (16.5–18.7)	**15.2% (13.3–17.2)**	**9.7% (8.2–11.5)**	14.9% (11.2–19.9)	11.1% (6.6–18.5)	**10.1% (9.0–11.2)**	**6.9% (5.5–8.6)**
	(18,321 observations deleted due to missingness)		(5573 observations deleted due to missingness)		(693 observations deleted due to missingness)		(7707 observations deleted due to missingness)	
Radiotherapy								
2007–2009	**9.9% (8.8–11.0)**	**13.8% (12.8–14.9)**	7.7% (6.4–9.3)	10.2% (8.9–11.7)	7.8% (4.8–12.6)	10.9% (7.8–15.2)	7.9% (6.5–9.6)	6.4% (5.4–7.5)
2010–2012	**9.8% (8.9–10.8)**	**12.7% (12.0–13.6)**	9.3% (7.9–10.9)	8.5% (7.4–9.7)	8.3% (5.5–12.6)	7.9% (5.7–10.8)	6.9% (5.8–8.3)	5.6% (4.8–6.5)
2013–2015	11.9% (10.9–12.9)	13.5% (12.8–14.3)	8.1% (6.8–9.6)	10.0% (8.9–11.3)	7.1% (4.4–11.6)	5.7% (3.7–8.6)	**8.5% (7.3–9.9)**	**5.4% (4.7–6.3)**
2016–2018	16.4% (15.1–17.8)	18.6% (17.6–19.6)	12.7% (10.7–15.2)	11.8% (10.3–13.4)	10.0% (5.9–17.1)	13.7% (9.9–19.0)	10.6% (9.0–12.6)	8.6% (7.6–9.8)
	(20,938 observations deleted due to missingness)		(6267 observations deleted due to missingness)		(794 observations deleted due to missingness)		(9405 observations deleted due to missingness)	
Immunotherapy								
2007–2009	–	–	–	–	–	–	–	–
2010–2012	–	–	–	–	–	–	–	–
2013–2015	–	–	–	–	–	–	–	–
2016–2018	**22.7% (20.5–25.2)**	**16.6% (15.7–17.6)**	**15.2% (11.0–20.9)**	**11.7% (10.3–13.2)**	–	–	–	–
	(29,800 observations deleted due to missingness)		(8782 observations deleted due to missingness)					

NSCLC and SCLC patients, across all four time-periods, demonstrated an increased 3-year survival rate following chemotherapy. In fact, both NSCLC and SCLC patients experienced an average increase of 2.3 percentage-points in their respective 3-year survival rate when treated with chemotherapy.

Minimal to no increase in 3-year survival was shown in NSCLC patients when receiving radiotherapy.

In contrast, SCLC patients benefited from receiving radiotherapy, although increases in 3-year survival were modest when compared to the increase shown by chemotherapy treatment.

Since immunotherapy was only fully incorporated in therapy regimes starting in 2016/2017, we only analyzed the immunotherapy data for the last time-period. NSCLC patients that received immunotherapy showed an increase in 3-year survival by up to 6.1 percentage points (adenocarcinoma in the time-period 2016–2018).

Over time, the survival of both the therapy-received and no-therapy-received groups did rise.

### Survival analysis

The median survival time for patients diagnosed with primarily metastatic adenocarcinoma was 7.8 months (95% CI 7.8–7.8), squamous cell carcinoma was 6.9 months (95% CI 6.0–6.9), large cell carcinoma was 4.8 months (95% CI 4.8–5.8), and for patients diagnosed with SCLC the median survival time was 6.9 months (95% CI 6.9–7.8) (Table 3).

**Table 3:** Median and 3-year survival time of patients diagnosed with primarily metastatic lung cancer 2007–2018 (95% CI).

Characteristics	NSCLC adenocarcinoma	NSCLC squamous cell carcinoma	NSCLC large cell carcinoma	SCLC
**Median OS, months (95% CI)**	7.8 (7.8–7.8)	6.9 (6.0–6.9)	4.8 (4.8–5.8)	6.9 (6.9–7.8)
2007–2009	7.8 (7.8–7.8)	6.9 (6.9–6.9)	4.8 (4.8–6.0)	6.9 (6.9–7.8)
2010–2012	7.8 (6.9–7.8)	6.9 (6.0–6.9)	4.8 (4.8–5.8)	7.8 (6.9–7.8)
2013–2015	6.9 (6.9–7.8)	6.0 (6.0–6.9)	4.8 (4.8–5.8)	6.9 (6.9–6.9)
2016–2018	7.8 (7.8–7.8)	6.9 (6.0–6.9)	6.0 (5.8–6.9)	7.8 (6.9–7.8)
**3-year survival, % (95% CI)**	13.4% (13.1–13.7)	9.5% (9.0–9.9)	8.0% (7.0–9.1)	6.4% (6.1–6.7)
2007–2009	12.1% (11.5–12.8)	8.7% (7.9–9.6)	7.5% (5.8–9.8)	6.2% (5.6–6.9)
2010–2012	11.4% (10.9–11.9)	8.5% (7.8–9.3)	8.4% (6.8–10.4)	5.7% (5.2–6.3)
2013–2015	12.8% (12.3–13.2)	9.1% (8.3–9.8)	5.5% (4.1–7.4)	6.0% (5.5–6.5)
2016–2018	17.1% (16.5–17.8)	12.0% (11.0–13.1)	11.8% (9.2–15.2)	8.1% (7.4–8.8)

While the median OS did not rise for NSCLC adenocarcinoma or squamous cell carcinoma; it rose marginally for NSCLC large cell cancer patients and SCLC patients, approximately 5 weeks and 4 weeks respectively.

The 3-year survival did indeed rise significantly if compared to the earliest time-period for all lung cancer types except for the NSCLC large cell carcinoma in the last time-period.

The Kaplan–Meier plots (Table 4) show the 3-year survival of the patients with primarily metastatic lung cancer included in this study.

**Table 4:** Kaplan–Meier plots showing the 3-year survival of patients diagnosed with primarily metastatic lung cancer 2007–2018, separated by sex and histology of the NSCLC.

	Female patients	Male patients
NSCLC adenocarcinoma		
NSCLC squamous cell carcinoma		
NSCLC large cell carcinoma		
SCLC		

Both NSCLC and SCLC patients show the longest survival in the most recent time-period.

The differences in survival between the four time-periods of NSCLC patients were significant (log-rank test, p < 0.0001), while the survival curves for the female SCLC patients were nearly identical (log-rank test, p = 0.22).

The lower survival of NSCLC patients for the first three time-periods compared to the most recent time-period (2016–2018) was most noticeable after approximately 50 weeks.

Overall, it can be observed that the biggest increase in survival for both female and male NSCLC patients is in the time between 2016 and 2018.

The hazard ratios of the considered study population are shown in Table 5. A more detailed version of Table 5 can be found in the appendix (Table S9).

**Table 5:** Hazard ratios (95% confidence interval in brackets) illustrating the survival of patients diagnosed with primarily metastatic lung cancer 2007–2018; significant results are portrayed in bold numbers.

	NSCLC		SCLC	
	Male	Female	Male	Female
After adjusting for time-periods (2007–2009 ref)				
2010–2012	1.02 (1.00–1.05)	1.01 (0.97–1.05)	1.00 (0.95–1.04)	0.99 (0.93–1.06)
2013–2015	1.01 (0.99–1.04)	1.01 (0.98–1.05)	1.04 (1.00–1.09)	1.01 (0.95–1.08)
2016–2018	**0.91 (0.89–0.94)**	**0.91 (0.88**–**0.95)**	0.97 (0.93–1.01)	0.96 (0.90–1.02)
After adjusting for age and time-periods				
2010–2012	1.02 (0.99–1.04)	1.00 (0.97–1.04)	0.97 (0.93–1.02)	0.98 (0.92–1.05)
2013–2015	1.00 (0.97–1.02)	0.99 (0.96–1.03)	1.01 (0.96–1.05)	0.99 (0.93–1.06)
2016–2018	**0.90 (0.87**–**0.92)**	**0.89 (0.86**–**0.92)**	**0.93 (0.89**–**0.98)**	**0.92 (0.86**–**0.98)**
After adjusting for age, time-periods and therapies (chemo-, radio-, immunotherapy)				
2010–2012	**1.05 (1.01**–**1.09)**	1.00 (0.95–1.06)	1.02 (0.96–1.07)	0.96 (0.88–1.04)
2013–2015	**1.05 (1.02**–**1.09)**	1.05 (1.00–1.11)	1.03 (0.98–1.09)	0.98 (0.90–1.07)
2016–2018	**0.95 (0.91**–**0.98)**	**0.94 (0.89**–**0.99)**	**0.94 (0.88**–**0.99)**	**0.88 (0.81**–**0.96)**
After adjusting for age, time-periods, therapies (chemo-, radio-, immunotherapy) and TNM-Status (T1–T4 and N0–N3)				
2010–2012	**1.06 (1.02**–**1.10)**	1.03 (0.96–1.10)	1.05 (0.98–1.13)	0.92 (0.83–1.02)
2013–2015	**1.08 (1.03**–**1.12)**	1.07 (1.00–1.14)	1.07 (1.00–1.15)	0.99 (0.89–1.09)
2016–2018	**0.94 (0.90**–**0.99)**	0.96 (0.89–1.02)	0.96 (0.90–1.03)	**0.84 (0.76**–**0.93)**

A decrease in HRs for the last time-period compared to the first time-periods can be observed for all analyzed types of primarily metastatic lung cancers. That effect is significant, though it correlates to only a small advantage in survival. It also remains observable after adjusting for all the chosen confounders. No significant model improvement was shown when an interaction term was added to the model.

NSCLC patients, after adjusting, benefited the most from receiving chemotherapy (HR = 0.70 [95% CI 0.68–0.72] for male, 0.74 [95% CI 0.72–0.78] for female patients) and immunotherapy (HR = 0.70 [95% CI 0.66–0.74] for male, 0.66 [95% CI 0.62–0.71] for female patients) when compared to no treatment. Female patients diagnosed with squamous cell carcinoma have a 1.38 higher chance of death when compared to adenocarcinoma patients; similar numbers are shown for male patients (HR = 1.23 [95% CI 1.15–1.31]).

Both male and female patients benefited from receiving chemotherapy and radiotherapy.

Female SCLC patients show only after adjusting for age, a significant HR for the last time-period (when compared to the first time-period).

### Bias and sensitivity analysis

The comparison of the HRs from West Germany with the HRs from East Germany showed no significant differences. The HRs proved themselves to be relatively robust.

The proportion of stage IV lung cancer patients, when compared to all lung cancer patients in the data set, slowly and steadily rose over time during the course of this study, as expected. Whereas in 2007, at the beginning of the time period studied, the percentage of this patient group was 42.9%, by 2018, at the end of the study, it rose about 6 percentage points to 49.5% (Table 6).

**Table 6:** Percentage of stage IV lung cancer patients from all patients in the RKI data set over time.

Year of diagnosis	Percentage of stage lV lung cancer patients (%)
2007	42.90
2008	43.68
2009	45.79
2010	46.76
2011	47.54
2012	47.85
2013	49.03
2014	49.17
2015	49.40
2016	50.79
2017	49.98
2018	49.48

When checking for interactions between the different therapies we found a significant interaction between chemotherapy and immunotherapy for NSCLC patients. The combined effect of both therapies results in a hazard ratio of 1.5, indicating that the risk of death increases by approximately 53.2%. For SCLC patients, the combined use of chemotherapy and radiotherapy results in a hazard ratio of 0.88.

No other significant interactions between the different therapies were detected.

## Discussion

In summary, advancements in radiotherapy and the increasing application of it, as well as the application of chemotherapy and immunotherapy (in more recent years), lead to an increased survival time of patients diagnosed with lung cancer^20^. This can be shown by the outcome of this study, which indicates that as time progresses and therapies become more efficient, lung cancer patient survival rates also increase.

During the observed time, there was a continuous increase in the use of immunotherapies. The publication of a new version of the lung cancer treatment guidelines in Germany (S3-Leitlinie Prävention, Diagnostik, Therapie und Nachsorge des Lungenkarzinoms) in 2018 led to a more intensified use of immunotherapy treatments at the lung cancer UICC stage IV^6^. That increased use of personalized treatment can be interpreted as one of the major reasons for the prolonged survival in the time-period 2016–2018 in comparison to other time-periods.

NSCLC patients demonstrated minimal to no increase in 3-year survival rates following radiotherapy treatment. One explanation for this is that the standard therapy consists of chemotherapy, but not necessarily radiotherapy—this is especially exemplified in the fact that patients who receive radiotherapy often have symptomatic cancer growth, which correlates with a poor survival probability. That might lead to a biased situation; meaning that the patient group who receives radiotherapy is a patient group that already has a lower survival rate to begin with.

Another reason for inconsistent numbers is that the documentation of therapy regimes in the German cancer registries do not always happen in a standardized way.

SCLC patients in all four time-periods benefited from receiving radiation therapy, although it resulted in only a modest increase in 3-year survival when compared to the increase shown by chemotherapy treatment. This improvement of survival was also shown in an international study by the EORTC Radiation Oncology and Lung Cancer Groups^21^ for prophylactic cranial irradiation in extensive disease SCLC. In the EORTC study, SCLC patients show notably fewer symptomatic brain metastases and an improved survival rate when receiving cranial irradiation.

While the median OS did not rise for NSCLC adenocarcinoma or squamous cell carcinoma, it rose only marginally for NSCLC large cell cancer patients and SCLC patients. This shows that the survival time of most primarily metastatic patients did not rise substantially.

However, the 3-year Survival did indeed rise significantly if compared to the earliest time-period for all lung cancer types included in the study except for the NSCLC large cell carcinoma. This indicates that for certain patients, but not for the majority, survival can successfully be prolonged. This certain group might contain patients that respond well to new therapies, such as immunotherapies.

Overall, the rather low increase in survival can also be explained by the large sample size which includes all patients, without selection bias.

The 3-year survival rates rose over time for patients that received treatment as well as for patients that received no treatment. There are several possible reasons for that; one being the introduction of PET-CT leading to improved diagnostics, another being advancements in supportive therapy also leading to a prolonged survival for patients that were not suitable for chemotherapy or decided against it. As a limitation to the study, it is important to note that this could also be due to lower treatment effects than expected.

Only after adjusting for age, female SCLC patients show a significant HR for the last time-period (when compared to the first time-period). An underlying reason for this may be due to the fact that, in the most recent time-period, patients are generally older, so the HR is not significant before adjusting for age.

In all four time-periods there are more male patients diagnosed with primarily metastatic lung cancer. A large contribution to this outcome is smoking, which is one of the major risk factors for lung cancer—this is especially true for squamous cell carcinoma, which has a proven correlation to smoking in patients^22^. Although the rate of smoking in women is rising, men still remain the largest group of smokers in the German population and globally^23^.

The growing percentage of female smokers might explain the increased number of women diagnosed throughout the tracked time-periods. However, the highest percentage of female cases can be found in the adenocarcinoma group, which is the most common lung cancer in non-smokers^24^. Additionally, female cases showed overall lower HR ratios when compared to male patients. In our study, patients diagnosed with adenocarcinoma of the lung showed the longest 3-year survival.

The amount of diagnosed lung cancer grew progressively over time, apart from large cell carcinoma and squamous cell carcinoma. More recent data shows that there is a notable decline in the overall prevalence of smoking in Germany^25^. This decline contributes to a decreasing number of patients diagnosed with squamous cell carcinoma. Similar results can also be found in studies investigating the topic^26^. For large cell carcinoma, it can also be noted that the rather small sample size makes it difficult to estimate an effect from the differing proportions diagnosed over time.

The mean age of diagnosis increases by 0.7 years between 2007 and 2018 for all observed cancer types. This effect can be explained by the aging population of Germany: according to the federal office of statistics (Statistisches Bundesamt) in Germany, the largest population group is over 50 years of age, with every subsequent generation decreasing in number. This information reveals that these changes are not due to new diagnostic techniques, but rather to the general demographic changes in the population.

The newest version of the lung cancer treatment guidelines in Germany, (S3-Leitlinie Prävention, Diagnostik, Therapie und Nachsorge des Lungenkarzinoms) published in 2022, suggest screening of high risk patients for the first time in history; the effect on the patient population regarding the diagnosis of lung tumors is not yet known.

There is a notable discrepancy between the survival of primarily metastatic lung cancer patients in epidemiological studies (like this study) and clinical studies. For instance after the application of a PD-1 inhibitor the OS of stage IV lung cancer patients was found to be 22.2 months in a clinical study^27^. This contrast is primarily due to selection bias in clinical studies, where patients are often selected based on specific criteria that may exclude those with poorer prognoses or additional health complications. Consequently, clinical study populations may not fully represent the broader, more diverse patient population diagnosed with lung tumors in real-world settings. This selection bias can lead to better survival outcomes in clinical trials compared to epidemiological studies, where a wider range of patient conditions and treatment responses are captured.

The interaction between chemotherapy and immunotherapy in NSCLC patients is indicating an increased risk of death. Patients that were treated with both therapies could have had a worse prognosis resulting in a more intense therapy regime and possibly also a higher chance of toxicity or adverse effects.

Between the other therapies where we found no statistical effect, the impact of each treatment on patient survival operates independently rather than in combination.

### Strengths and limitations

The data for this study was extracted from respected and nationwide representative databases. The German registries are well structured and constantly used for data analysis, for example, to monitor cancer survival^28–30^. Furthermore, registry based studies are known to reflect the ‘real-world’ settings more fully.

Other studies have investigated the topic of new trends in lung cancer survival as well. In 2020, Howlader et al.^26^ published a study reporting on advances in the treatment of lung cancer in terms of population mortality, finding similar results to our study. While some demographic differences in Germany, when compared to the United States, determine the different outcomes, a major difference is that our analysis only investigated primarily metastatic lung cancer patients, allowing us to analyze this vulnerable patient group more precisely.

Our study also included the large sample size of 127,723 patients that were followed over a long period of 11 years. As stated above, we excluded registries with high DCO to ensure high data quality and we conducted sensitivity analyses to ensure that the use of both East and West German cancer registries did not confound our results.

Nevertheless, there are some limitations to this study.

Due to the fact that in the most recent years more federal states started to contribute to the cancer registries, the sample size for the more recent time-periods are thoroughly higher than in the first time-period. A possible consequence of this is a bias of period-related treatment effect.

Our study does not specifically include targeted therapies as a treatment option because the dataset provided by the RKI does not divide targeted therapies into a separately tracked treatment option. The use of targeted therapies is recorded under “other therapies”.

However, we do not expect a major bias of the general conclusion as the effect of targeted therapy would be part of the general effect of time on survival. By controlling for immunotherapy, chemotherapy and radiation we can presume that other treatments such as targeted therapy trigger this effect which was the primary objective of our study. Since we have adjusted for chemotherapy, radiotherapy, and immunotherapy, the remaining period effect on survival is expected to be caused by targeted therapies.

Nevertheless, it is possible that, especially in the early times of introducing these new advanced therapies, patients receiving targeted therapies were wrongfully added to the group of immunotherapy. In order to minimize the effect of this bias, as mentioned above, we withheld the survival analysis for immunotherapy in the first three time-periods.

The German Cancer Registries do not differentiate between thoracic radiation therapy and radiation therapy for metastases. Since our study only includes patients that are already metastatic at the time of diagnosis, any radiation therapy in that stage of lung cancer aims at reducing symptoms rather than prolonging the patient’s life. While we do acknowledge this as a limitation to our analysis, we believe that it does not noticeably affect the outcome since the goal of the study is to analyze the survival and not the symptomatic status of the patients.

Another limitation to our study concerns the database, which does not differentiate the subcategories of the ICD-O codes. For example, all patients with the adenocarcinoma of the lung have the ICD-O Code 8140. That code should also include the adenoma of the lung, which would wrongfully add to the analysis. Since the alveolar adenoma is a very rare benign tumor that only has been described about 40 times in literature, mostly in case reports^31^, the effects of this are negligible.

Moreover, this study, as it only uses observational data, is limited to the variables provided by the German cancer registries. It does not include additional variables associated with survival, for instance, the presence of comorbidities or the performance status of the patients. Due to the lack of these additional variables, there is a possibility of confounding by indication.

The analysis of the stage IV lung cancer patients over time, when compared to all patients in the data set, shows that there was a steady rise in the percentage of this certain patient group, which theoretically could confound results. In the earlier years, where there was less availability of the PET/CT for staging, patients could have been misclassified into lower stages. Especially those patients with only singular and small metastases can be treated with a curative approach^6^—that could lead to a biased situation, resulting in a better survival in the more recent time-periods, since earlier, those patients may not have ever been diagnosed as stage IV.

However, the effect is to be questioned since the percentage of diagnosed stage IV patients only increased marginally making confounding less likely.

## Conclusion

The analysis of the population-based data shows an increase in survival of all stage IV lung cancer patients, especially in the most recent time-period. For the first time in ten years there is an observable upsurge in the survival of this group of patients with an unfavorable prognosis. The extended use of immunotherapy for NSCLC patients, the continuous application of chemotherapy and the advancement in radiotherapy led to a rise in the 3-year survival, male and female, although the improvement is only to be valued as moderate. Female SCLC patients, after adjusting, had the biggest increase in survival. The group of male SCLC patients and female NSCLC patients showed the smallest increase in survival.

The results of this study indicate that there is a need to further increase the use of innovative and highly effective therapies, as well as a need for advanced research of more reliable therapies for this vulnerable group of patients.

### Supplementary Information

Supplementary Information.

## Data Availability

The data of this study was provided by the Robert Koch Institute (RKI). The authors do not own this data and hence are not permitted to share it in its original form (only in aggregate form, for instance in publications).
