# Supplementary material for: Population based study on the progress in survival of primarily metastatic lung cancer patients in Germany
Source: Sci Rep. 2024 Jul 11;14:16005. doi: 10.1038/s41598-024-66307-3 (PMC11239887; doi:10.1038/s41598-024-66307-3)
Supplement: Supplementary file 1 — Supplementary Information. [file 41598_2024_66307_MOESM1_ESM.docx]

**Appendix**

**Table 7** Lung cancer morphology groups included in the study

| Broad group | Specific group | ICD-O morphology code | Description (Lim et al. 2018) | Total patients (n= ) |
| --- | --- | --- | --- | --- |
| SCLC  NSCLC | Adenocarcinoma  Large cell carcinoma  Squamous cell carcinoma | 8002  8041  8042  8043  8044  8045  8050  8140  8141  8144  8190  8200  8201  8211  8230  8240  8244  8246  8249  8250  8251  8252  8253  8254  8255  8260  8263  8265  8310  8323  8480  8481  8490  8560  8570  8574  8576  8012  8013  8014  8031  8052  8070  8071  8072  8073  8074  8075  8078  8083  8084 | Malignant tumor, small cell type  Small cell carcinoma, NOS  Oat cell carcinoma  Small cell carcinoma, fusiform cell  Small cell carcinoma, intermediate cell  Combined small cell carcinoma  Papillary carcinoma, NOS Adenocarcinoma, NOS  Scirrhous adenocarcinoma  Adenocarcinoma, intestinal type  Trabecular adenocarcinoma  Bronchial adenoma, cylindroid  Cribriform carcinoma, NOS  Tubular adenocarcinoma  Solid adenocarcinoma with mucin formation  Bronchial adenoma, carcinoid  Mixed adenoneuroendocrine carcinoma  Neuroendocrine carcinoma, NOS  Atypical carcinoid tumor  Bronchiolo-alveolar adenocarcinoma, NOS  Alveolar adenocarcinoma  Bronchiolo-alveolar carcinoma, non- mucinous  Bronchiolo-alveolar carcinoma, mucinous  Bronchiolo-alveolar carcinoma, mixed mucinous and non-mucinous  Adenocarcinoma with mixed subtypes  Papillary adenocarcinoma, NOS  Adenocarcinoma in tubulovillous adenoma  Micropapillary carcinoma, NOS  Clear cell adenocarcinoma, NOS  Mixed cell adenocarcinoma  Mucinous adenocarcinoma  Mucin-producing adenocarcinoma  Signet ring cell carcinoma  Adenosquamous carcinoma  Adenocarcinoma with squamous metaplasia  Adenocarcinoma with neuroendocrine differentiation  Hepatoid adenocarcinoma  Large cell carcinoma, NOS  Large cell neuroendocrine carcinoma  Large cell carcinoma with rhabdoid phenotype  Giant cell carcinoma  Papillary squamous cell carcinoma  Squamous cell carcinoma, NOS  Squamous cell carcinoma, keratinizing, NOS  Squamous cell carcinoma, large cell, nonkeratinizing Squamous cell carcinoma, small cell, nonkeratinizing Squamous cell carcinoma, spindle cell  Squamous cell carcinoma, adenoid  Squamous cell carcinoma with horn formation  Basaloid squamous cell carcinoma  Squamous cell carcinoma, clear cell type | 46  24 028  1583  27  275  845  13  54 903  12  14  7  28  12  44  704  339  31  1727  253  838  28  34  106  12  426  777  40  55  85  36  552  590  118  1317  7  124  31  1559  1403  7  39  7  14 149  2949  3073  151  47  10  19  167  12 |

**Table 8** Characteristics of patients diagnosed with primarily metastatic lung cancer 2007-2018: Diagnosed histologies over time shown as percentages of the period’s total patients

| Characteristics | Total | Period 2007-2009 | Period 2010-2012 | Period 2013-2015 | Period 2016-2018 |
| --- | --- | --- | --- | --- | --- |
| All patients | 127 723 | 22 862 (100%) | 31 475 (100%) | 36 755 (100%) | 36 631 (100%) |
| SCLC patients | 26 804 | 5 029 (22%) | 6 617 (21%) | 7 666 (20.9%) | 7 492 (20.5%) |
| NSCLC patients | 95 060 | 16 564 (72.5%) | 23 413 (74.4%) | 27 576 (75%) | 27 507 (75.1%) |
| *- Adenocarcinoma* | *63 263* | *9 905 (44.3%)* | *15 290 (48.6%)* | *18 851 (51.3%)* | *19 217 (52.5%)* |
| *- Squamous cell carcinoma* | *20 584* | *4 326 (18.9%)* | *5 114 (16.2%)* | *5 708 (15.5%)* | *5 436 (14.8%)* |
| *- Large cell carcinoma* | *3 008* | *678 (3%)* | *895 (2.8%)* | *763 (2.1%)* | *672 (1.8%)* |
| Other histologies | 5 859 | 1 269 (5.5%) | 1 445 (4.6%) | 1 513 (4.1%) | 1 632 (4.4%) |

**Table 9** Detailed Hazard ratios (95% confidence interval in brackets) illustrating the survival of patients diagnosed with primarily metastatic lung cancer 2007-2018

|  | NSCLC |  | SCLC |  |
| --- | --- | --- | --- | --- |
|  | Male | Female | Male | Female |
| Time-periods: 2007-2009 *ref*  2010-2012  2013-2015  2016-2018 | 1.02 (1.00-1.05)  1.01 (0.99-1.04)  0.91 (0.89-0.94) | 1.01 (0.97 - 1.05)  1.01 (0.98 - 1.05)  0.91 (0.88 - 0.95) | 1.00 (0.95 - 1.04)  1.04 (1.00 - 1.09)  0.97 (0.93 - 1.01) | 0.99 (0.93 - 1.06)  1.01 (0.95 - 1.08)  0.96 (0.90 - 1.02) |
| Time-periods: 2007-2009 *ref*  2010-2012  2013-2015  2016-2018  Age | 1.02 (0.99 - 1.04)  1.00 (0.97 - 1.02)  0.90 (0.87 - 0.92)  1.17 (1.16 - 1.18) | 1.00 (0.97 - 1.04)  0.99 (0.96 - 1.03)  0.89 (0.86 - 0.92)  1.17 (1.16 - 1.18) | 0.97 (0.93 - 1.02)  1.01 (0.96 - 1.05)  0.93 (0.89 - 0.98)  1.24 (1.22 - 1.26) | 0.98 (0.92 - 1.05)  0.99 (0.93 - 1.06)  0.92 (0.86 - 0.98)  1.28 (1.25 - 1.31) |
| Time-periods: 2007-2009 *ref*  2010-2012  2013-2015  2016-2018  Age  Therapy: without therapy *ref*  Chemotherapy  Radiotherapy  Immunotherapy | 1.05 (1.01 - 1.09)  1.05 (1.02 - 1.09)  0.95 (0.91 - 0.98)  1.13 (1.12 - 1.15)  0.72 (0.70 - 0.74)  1.05 (1.02 - 1.07)  0.69 (0.65 - 0.72) | 1.00 (0.95 - 1.06)  1.05 (1.00 - 1.11)  0.94 (0.89 - 0.99)  1.14 (1.13 - 1.16)  0.77 (0.75 - 0.80)  1.18 (1.14 - 1.23)  0.66 (0.62 - 0.70) | 1.02 (0.96 - 1.07)  1.03 (0.98 - 1.09)  0.94 (0.88 - 0.99)  1.20 (1.18 - 1.23)  0.61 (0.58 - 0.64)  0.81 (0.78 - 0.84) | 0.96 (0.88 - 1.04)  0.98 (0.90 - 1.07)  0.88 (0.81 - 0.96)  1.21 (1.18 - 1.25)  0.68 (0.64 - 0.73)  0.81 (0.76 - 0.86) |
| Time-periods: 2007-2009 *ref*  2010-2012  2013-2015  2016-2018  Age  Therapy without therapy *ref*  Chemotherapy  Radiotherapy  Immunotherapy  TNM-status: T1 *ref*  T2  T3  T4  N0 *ref*  N1  N2  N3 | 1.06 (1.02 - 1.10)  1.08 (1.03 - 1.12)  0.94 (0.90 - 0.99)  1.14 (1.13 - 1.16)  0.70 (0.68 - 0.72)  1.06 (1.03 - 1.09)  0.70 (0.66 - 0.74)  1.18 (1.12 - 1.25)  1.29 (1.22 - 1.36)  1.40 (1.33 - 1.48)  1.32 (1.25 - 1.40)  1.50 (1.43 - 1.57)  1.68 (1.61 - 1.76) | 1.03 (0.96 - 1.10)  1.07 (1.00 - 1.14)  0.96 (0.89 - 1.02)  1.15 (1.12 - 1.17)  0.74 (0.72 - 0.78)  1.17 (1.12 - 1.23)  0.66 (0.62 - 0.71)  1.22 (1.13 - 1.32)  1.38 (1.27 - 1.49)  1.45 (1.34 - 1.56)  1.39 (1.29 - 1.51)  1.51 (1.42 - 1.61)  1.84 (1.72 - 1.96) | 1.05 (0.98 - 1.13)  1.07 (1.00 - 1.15)  0.96 (0.90 - 1.03)  1.19 (1.16 - 1.22)  0.63 (0.60 - 0.66)  0.80 (0.76 - 0.84)  1.14 (1.03 - 1.27)  1.17 (1.06 - 1.31)  1.22 (1.11 - 1.35)  1.10 (0.96 - 1.25)  1.15 (1.02 - 1.29)  1.31 (1.17 - 1.46) | 0.92 (0.83 - 1.02)  0.99 (0.89 - 1.09)  0.84 (0.76 - 0.93)  1.21 (1.17 - 1.26)  0.68 (0.63 - 0.74)  0.80 (0.75 - 0.86)  1.13 (0.97 - 1.31)  1.22 (1.05 - 1.42)  1.32 (1.16 - 1.52)  1.04 (0.87 - 1.25)  1.04 (0.90 - 1.21)  1.14 (0.98 - 1.32) |
